# TNFR2 unlocks a RIPK1 kinase activity-dependent mode of proinflammatory TNFR1 signaling

**DOI:** 10.1038/s41419-018-0973-3

**Published:** 2018-09-11

**Authors:** Daniela Siegmund, Martin Ehrenschwender, Harald Wajant

**Affiliations:** 1 Division of Molecular Internal Medicine, Department of Internal Medicine II, University Hospital Würzburg, Auvera Haus, Grombühlstraße 12, 97070 Würzburg, Germany; 2 Institute of Clinical Microbiology and Hygiene, University Hospital Regensburg, Franz-Josef-Strauss-Allee 11, 93053 Regensburg, Germany

## Abstract

TNF is not only a major effector molecule of PAMP/DAMP-activated macrophages, but also regulates macrophage function and viability. We recently demonstrated that TNFR2 triggers necroptosis in macrophages with compromised caspase activity by two cooperating mechanisms: induction of endogenous TNF with subsequent stimulation of TNFR1 and depletion of cytosolic TRAF2-cIAP complexes. Here we show that TNFR2 activation in caspase-inhibited macrophages results in the production of endogenous TNF and TNFR1 stimulation followed by upregulation of A20, TRAF1, IL-6, and IL-1β. Surprisingly, TNFR1-mediated induction of IL-6 and IL-1β was clearly evident in response to TNFR2 stimulation but occurred not or only weakly in macrophages selectively and directly stimulated via TNFR1. Moreover, TNFR2-induced TNFR1-mediated gene induction was largely inhibited by necrostatin-1, whereas upregulation of A20 and TRAF1 by direct and exclusive stimulation of TNFR1 remained unaffected by this compound. Thus, treatment with TNFR2/ZVAD enables TNFR1 in macrophages to stimulate gene induction via a pathway requiring RIPK1 kinase activity. TNFR2/ZVAD-induced production of IL-6 and IL-1β was largely blocked in necroptosis-resistant MLKL- and RIPK3-deficient macrophages, whereas induction of A20 and TRAF1 remained unaffected. In sum, our results show that in caspase-inhibited macrophages TNFR2 not only triggers TNF/TNFR1-mediated necroptosis but also TNF/TNFR1-mediated RIPK3/MLKL-dependent and -independent gene induction.

## Introduction

Macrophages can be activated by a variety of pathogen- and damage-associated molecular patterns (PAMPs and DAMPs) and are of major importance for innate immunity and tissue repair^1,2^. Activated macrophages not only act as phagocytes of pathogens and dead cells but also connect innate immunity with adaptive immunity by antigen presentation to T-helper cells and release of pleiotropic cytokines such as interferons and tumor necrosis factor (TNF). The latter is not only a major effector molecule of activated macrophages but also retroacts on this cell type resulting in enhanced and sustained activation. Dependent on the circumstances, TNF can also elicit cytotoxic effects in macrophages^3–15^.

TNF is initially expressed as a type II transmembrane protein. A soluble form of TNF furthermore originates from transmembrane TNF by proteolytic processing^16^. Both forms of TNF bind to two receptors of the TNF receptor superfamily (TNFRSF), TNFR1 and TNFR2. Although binding of soluble TNF by TNFR1 is fully sufficient for receptor activation, soluble TNF poorly stimulates TNFR2 signaling despite high-affinity binding^16^. In contrast, membrane TNF activates TNFR1 and TNFR2 with high efficiency. TNFR1 and TNFR2 are representatives of two distinct subgroups of the TNFRSF. TNFR1 is a member of the death receptor subgroup with the ability to stimulate apoptotic and/or necroptotic signaling^17,18^. However, TNFR1-induced cytotoxic signaling is normally masked by TNFRSF1A associated via death domain (TRADD)-mediated recruitment of TRAF2-cIAP1 and TRAF2-cIAP2 complexes, and the ability of TNFR1 to stimulate the classical nuclear factor-κB (NFκB) pathway, which controls the expression of various anti-apoptotic proteins^16–18^. TNFR2 belongs to the TRAF-interacting subgroup of the TNFRSF. The receptors of this subgroup are characterized by the ability to directly recruit TRAF adapter proteins, especially TRAF2, by virtue of short peptide motifs^19^. TNFR2 activates the classical and alternative NFκB pathway, but in contrast to TNFR1 it is not directly linked to cytotoxic pathways^14,16,20^. Interestingly, TNFR2 elicits cytotoxic activity by two indirect mechanisms: first, by triggering NFκB-dependent production of TNF and subsequent activation of the death receptor TNFR1, and second by depletion/degradation of the anti-cytotoxic TRAF2-cIAP1 and TRAF2-cIAP2 complexes, and limiting their availability for TNFR1^16^. Indeed, early on, we were able to show that TNFR2 enhances TNFR1-induced activation of caspase-8 and apoptosis^16^ and in recent years we and others showed that TNFR2-induced TRAF2-cIAP1/2 depletion also sensitizes for death receptor-induced necroptosis^10,14^.

Macrophages express TNFR1 as well as TNFR2, and are important producers of TNF upon exposure to PAMPs and DAMPs. In addition, both TNF receptors are able to induce the production of TNF in macrophages. In view of this complex interplay, it is not surprising that the molecular mechanisms of TNF signaling in macrophages are still poorly understood. We recently showed that TNFR2 induces necroptosis in macrophages compromised for caspase-8 activation by autocrine TNF production along with depletion/degradation of TRAF2-cIAP1/2 complexes^14^.

Here we investigated the effect of TNFR2 stimulation on NFκB-regulated factors. In contrast to TNFR1, TNFR2 induced NFκB-regulated genes preferentially when caspase activation was inhibited. Similar to TNFR2-induced necroptosis, TNFR2-induced gene induction largely occurred via endogenous TNF and subsequent TNFR1 stimulation in an RIPK1 kinase inhibitor-sensitive manner. Intriguingly, although TNFR2-induced interleukin (IL)-6 and IL-1β induction were co-mediated with necroptosis by RIPK3 and MLKL, the upregulation of TRAF1 and A20 bifurcated from necroptotic signaling upstream of RIPK3 but downstream of TNFR1-induced RIPK1 phosphorylation. Thus, in caspase-inhibited macrophages, TNFR2 unmasks a yet not recognized TNFR1-associated NFκB signaling pathway requiring RIPK1 kinase activity.

## Results

### Necrostatin-1 prevents gene induction by TNFR2 in caspase-inhibited macrophages

**Fig. 1:**
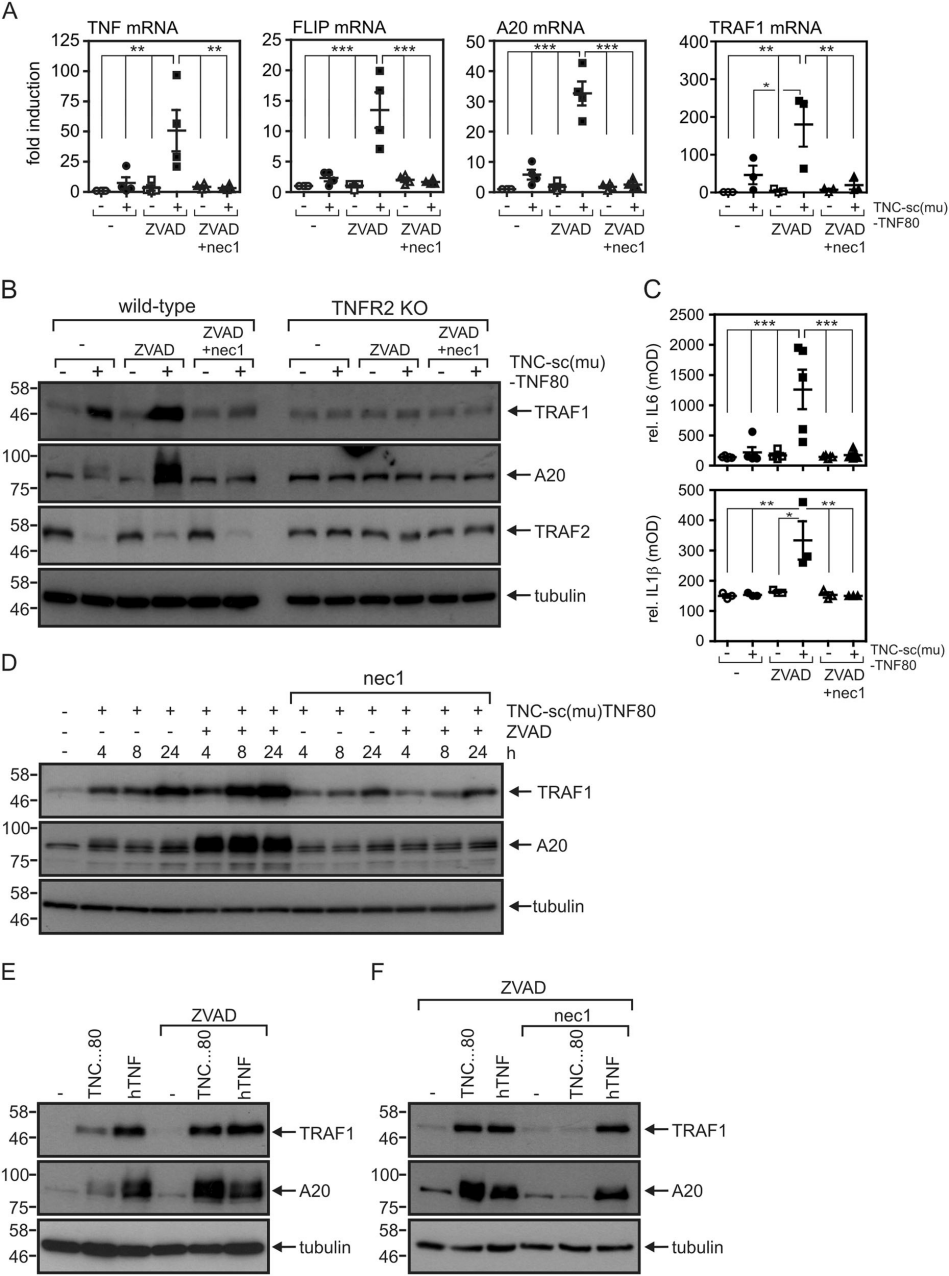
TNFR2-induced upregulation of NFκB-regulated factors is inhibited by necrostatin-1 in caspase-inhibited macrophages. **a** Macrophages were stimulated with the indicated mixtures of TNC-sc(mu)TNF80 (TNC…80, 200 ng/ml), ZVAD (Z, 20 µM), and necrostatin-1 (N, 45 µM). Next day, mRNA was isolated and expression of *Tnf, Cflar*, *A20*, and *Traf*1 were determined by qPCR. Shown are the mean ± SEM of four (TNF, FLIP, A20) or three (TRAF1) independent experiments. *p < 0.05, **p < 0.005, ***p < 0.001. **b** Wild-type and TNFR2-knockout macrophages were stimulated as indicated for 7 h and analyzed by western blotting. **c** Macrophages were stimulated for 36 h with combinations of TNC-sc(mu)TNF80, ZVAD (Z), and necrostatin-1 (N). IL-1β and IL-6 levels were quantified in the supernatants using ELISA assay. Shown are the mean ± SEM of five (IL-6) or three (IL-1β) independent experiments. *p < 0.05, **p < 0.005, ***p < 0.001. **d** Untreated and ZVAD-treated macrophages were stimulated for the indicated times with 200 ng/ml TNC-sc(mu)TNF80 in the absence and presence of necrostatin-1 (45 µM). Whole-cell lysates were analyzed by western blotting. **e** Untreated and ZVAD-treated macrophages were stimulated with 100 ng/ml human TNF (selective for murine TNFR1) or 200 ng/ml TNC-sc(mu)TNF80. Next day, total cell lysates were prepared for western blot analysis. **f** Western blot analysis of zVAD-fmk-treated macrophages, which were selectively stimulated overnight via TNFR1 (100 ng/ml human TNF) and TNFR2 (200 ng/ml TNC-sc(mu)TNF80) in the presence and absence of necrostatin-1 (45 µM)

To analyze the mechanisms of TNFR2-triggered gene induction in caspase-inhibited macrophages, we evaluated TNFR2-mediated induction of TNF and other NFκB-regulated factors at the mRNA level. For selective activation of TNFR2, we used TNC-sc(mu)TNF80, a fusion protein of the small trimerization domain of tenascin-C and three amino acid linker connected protomers of a TNFR2-specific mutant of soluble mouse TNF^21^. TNC-sc(mu)TNF80 is not only highly specific for TNFR2 but, due to its oligomeric nature, also overcomes the poor activity of soluble TNF trimers on TNFR2^21^. Unexpectedly, inhibition of RIPK1 kinase activity using necrostatin-1 strongly reduced TNC-sc(mu)TNF80-induced TNFR2-mediated transcription of the NFκB target genes *Tnf*, *Cflar*, *A20*, and *Traf1* in the presence of carbobenzoxy-valyl-alanyl-aspartyl-[O-methyl]-fluoromethylketone (ZVAD) (Fig. 1a) Similarly, ZVAD/necrostatin-1 treatment inhibited TNFR2-induced upregulation of A20, IL-6, and IL-1β at the protein level (Fig. 1b, c), whereas TNFR2-induced depletion of TRAF2 was expectedly unaffected (Fig. 1b). Loss of RIPK1 kinase activity also diminished TNFR2-induced TRAF1 expression, although at later time points a significant residual necrostatin-1-insensitive TRAF1 induction was observable (Fig. 1d). Selective activation of TNFR1 with human TNF^22^ also triggered expression of A20 and TRAF1. In contrast to TNFR2-induced gene induction, however, caspase inhibition and RIPK1 kinase activity were both dispensable (Fig. 1e, f). Collectively, our results suggest that TNFR2-induced upregulation of NFκB target genes in macrophages depends on (a) the absence of caspase activity and (b) functional RIPK1 kinase activity. The later is unexpected, as a direct role for RIPK1 in TNFR2 signaling has thus far not been established.

### Gene induction by TNFR2 in caspase-inhibited macrophages is largely mediated by endogenous TNF and TNFR1

**Fig. 2:**
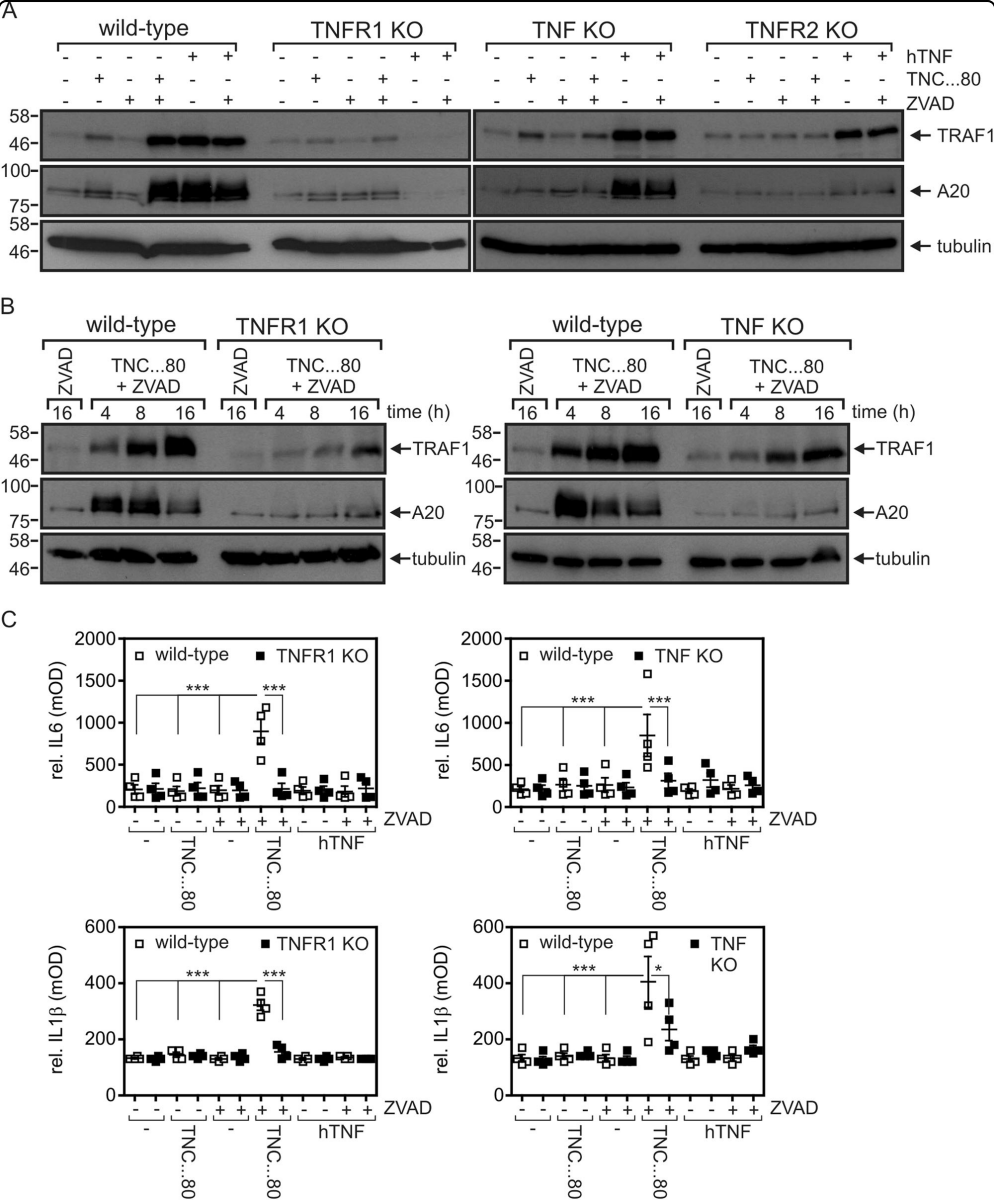
TNFR2-triggered gene induction is mediated by endogenous TNF and TNFR1 stimulation. **a** Wild-type, TNF-, TNFR1-, and TNFR2-knockout macrophages were challenged overnight as indicated with human TNF (100 ng/ml), TNC-sc(mu)TNF80 (200 ng/ml), and ZVAD (20 µM). Total cell lysates were analyzed by western blotting. **b** Wild-type, TNFR1-, and TNF-knockout macrophages were challenged with TNC-sc(mu)TNF80 (200 ng/ml)/ZVAD (20 µM) for the indicated times and total cell lysates were again analyzed by western blotting. **c** Macrophages were stimulated for 36 h as indicated with TNC-sc(mu)TNF80 and human TNF in the absence and presence of ZVAD. Supernatants were analyzed by ELISA assay with respect to their IL-1β and IL-6 content. Shown are the mean ± SEM of four independent experiments. *p < 0.05, ***p < 0.001

We previously showed that TNFR2 activation in caspase-inhibited macrophages can trigger RIKP1-dependent necroptosis in an auto-/paracrine manner via the TNF/TNFR1 axis^14^. Thus, TNF and TNFR1 may also have a key role in TNFR2-induced gene transcription in caspase-inhibited macrophages. Deficiency in TNF or TNFR1 abrogated TNFR2-induced expression of A20 (Fig. 2a, b) and significantly reduced upregulation of IL-6, IL-1β, and TRAF1 (Fig. 2a–c). Notably, a significant TNF/TNFR1-independent component of TRAF1 induction was observable (Fig. 2a, b). This is in good accordance with our earlier studies in macrophages showing activation of the alternative NFκB pathway by TNFR2^14^ and the well-established fact that TRAF1 is induced by both the classical and alternative NFκB pathway. Thus, the gene inductive effects of TNFR2 in macrophages are largely but not fully dependent on TNFR1. TNF deficiency did not affect TNFR1-mediated A20 and TRAF1 expression (Fig. 2a). Intriguingly, exclusive TNFR1 stimulation with human TNF failed to upregulate IL-6 and IL-1β (Fig. 2c). Mechanistically, these data suggest a crucial involvement of two at least partly different signaling pathways in TNFR2/ZVAD-triggered vs. TNFR1-mediated gene induction.

### Gene induction by TNFR2 in caspase-inhibited macrophages is largely independent from RIPK3 and MLKL

**Fig. 3:**
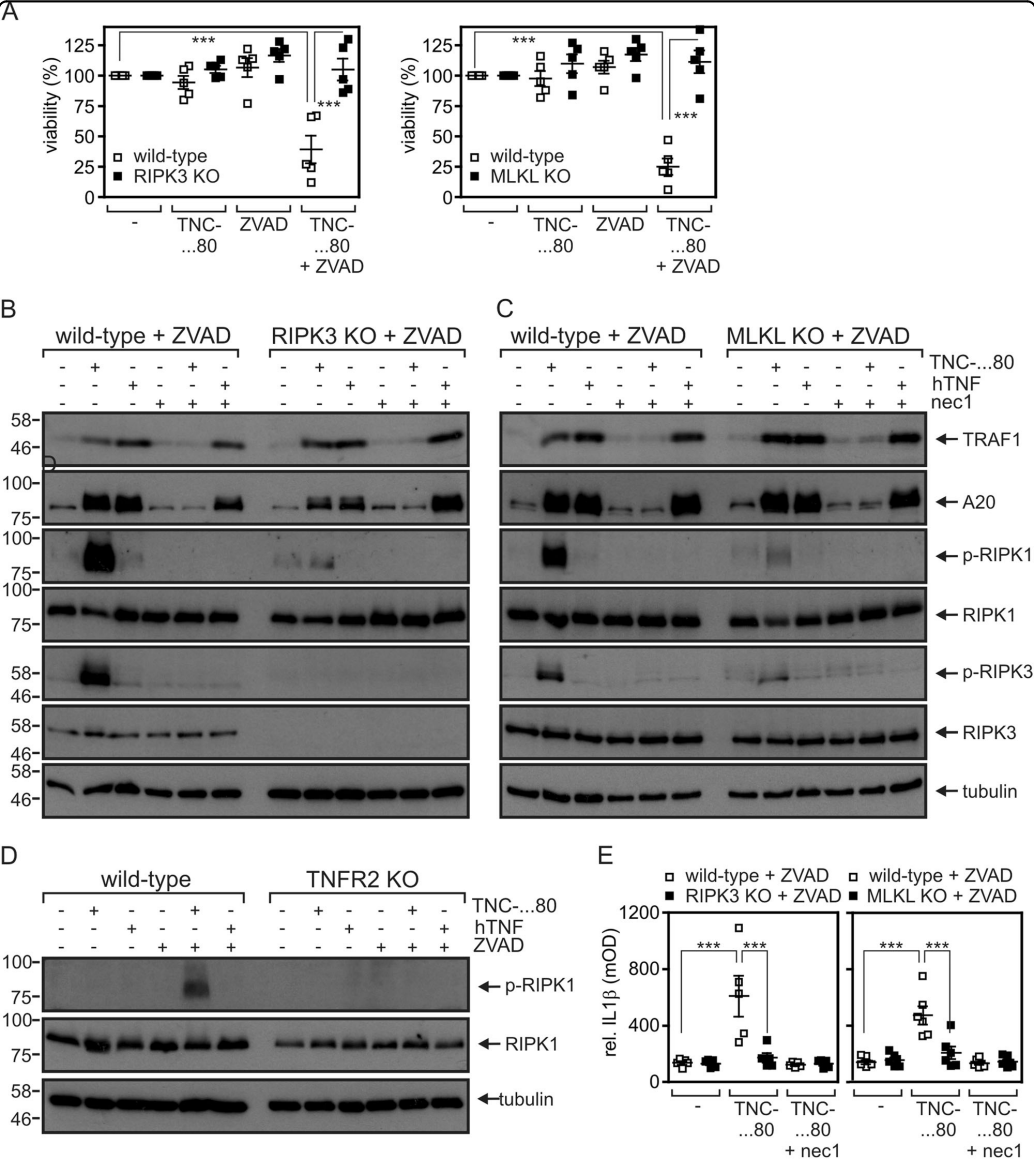
TNFR2-induced upregulation of A20 and TRAF1 is not an epiphenomenon of TNFR2-triggered necroptosis. **a** Wild-type, RIPK3-, and MLKL-knockout macrophages were stimulated as indicated with human TNF (100 ng/ml), TNC-sc(mu)TNF80 (200 ng/ml), and ZVAD (20 µM). Cell viability was quantified after 36 h using MTT. Data points of five independent experiments were shown with mean ± SEM (***p < 0.001). **b**, **c** RIPK3- (**b**) and MLKL- (**c**) knockout macrophages along with wild-type macrophages were treated overnight with the indicated combinations of 200 ng/ml TNC-sc(mu)TNF80, 100 ng/ml human TNF, 20 µM ZVAD, and necrostatin-1 (45 µM). Total cell lysates were prepared and analyzed by western blotting. **d** Wild-type and TNFR2-knockout macrophages were stimulated for 7 h with human TNF (100 ng/ml) or TNC-sc(mu)TNF80 (200 ng/ml) in the presence and absence of ZVAD (20 µM), and total cell lysates were analyzed by western blotting for RIPK1 phosphorylation. **e** The various macrophage types were stimulated for 36 h with the indicated mixtures of TNC-sc(mu)TNF80 (200 ng/ml), ZVAD (20 µM), and necrostatin-1 (45 µM). IL-1β in the supernatants was determined by ELISA assay. Shown are the mean ± SEM of five or six independent experiments. ***p < 0.001

In TNFR1 signaling, the kinase activity of RIPK1 is dispensable for NFκB activation but essential for necroptosis^17,18^. Accordingly, necrostatin-1 efficiently inhibits the later, whereas TNFR1-induced gene expression remains unaffected. We confirmed that RIPK1 kinase activity is dispensable for gene induction upon direct TNFR1 activation in macrophages (Figs. 1f, 3b, c). Indeed, in accordance with the fact that only exogenous activation of TNFR2, but not TNFR1 stimulation, induces necroptosis^14^, only TNC-sc(mu)TNF80 but not human TNF triggered RIPK1 phosphorylation (Fig. 3d). TNFR1 activation via TNFR2-triggered production of endogenous TNF, however, was significantly diminished in the presence of necrostatin-1 as is evident from the results shown in Figs. 1 and 2. One possible explanation for the different necrostatin-1 sensitivity is that the TNFR2-triggered mode of gene induction is associated with its ability to initiate TNF–TNFR1-dependent necroptosis^14^. To clarify this possibility, we investigated RIPK3- and MLKL-knockout macrophages. As expected, loss of RIPK3 or MLKL completely rescued macrophages from TNFR2/ZVAD-induced necroptosis (Fig. 3a). Surprisingly, however, TNFR2/ZVAD-induced A20 and TRAF1 expression was not significantly affected in caspase-inhibited RIPK3- and MLKL-knockout macrophages and still sensitive to necrostatin-1 (Fig. 3b, c). In contrast, TNFR2/ZVAD-induced production of IL-1β was severely reduced (Fig. 3e). This supports again the involvement of two at least partly different signaling pathways in TNFR2/ZVAD-triggered, TNFR1-mediated gene induction.

**Fig. 4:**
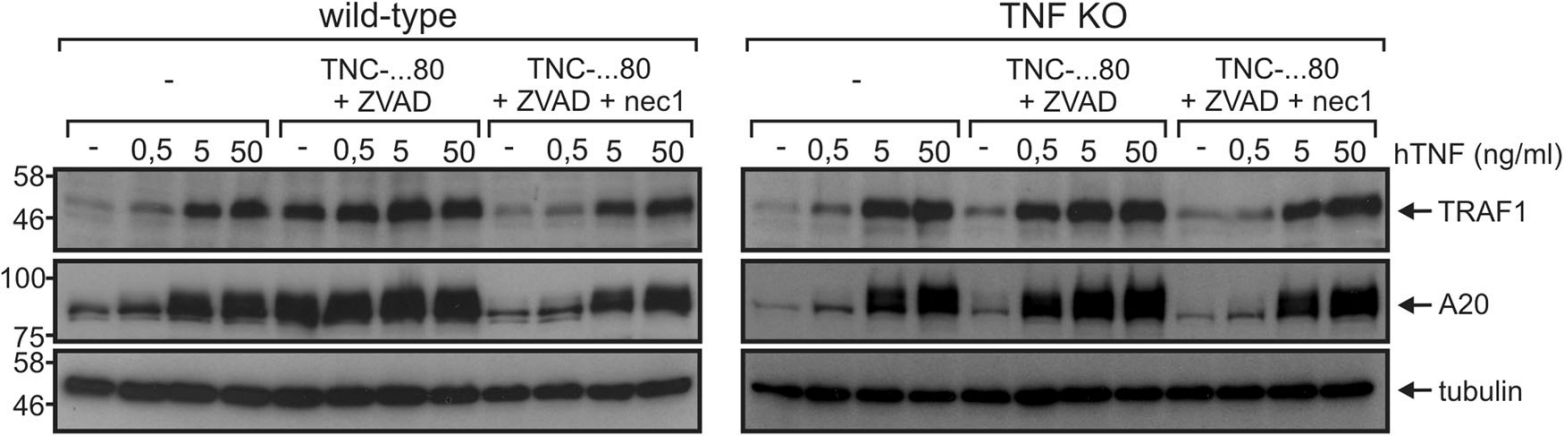
TNFR2 enables TNFR1 to induce TRAF1 and A20 via a necrostatin-1-sensitive pathway. Wild-type and TNF-knockout macrophages were stimulated as indicated with 200 ng/ml TNC-sc(mu)TNF80 and increasing concentrations of hTNF for 7 h and total lysates were analyzed by western blotting

Human TNF, irrespective of the presence of ZVAD, as well as TNFR2 stimulation in the presence of ZVAD resulted in TNFR1-dependent upregulation of A20 and TRAF1 (Fig. 2b). TNFR1 is the common signaling “bottleneck”, but only TNFR2/ZVAD-induced gene induction is inhibited by the RIPK1 kinase activity inhibitor necrostatin-1 (Figs. 1f, 3b). Potentially, TNFR2 unmasks in caspase-inhibited macrophages a TNFR1-triggered, TRAF1/A20-inducing RIPK1 kinase activity-dependent pathway, whose activity becomes not evident upon selective TNFR1 stimulation. To proof this hypothesis, we investigated TNFR1–TNFR2 cooperation in TNF-knockout cells. In this experimental setup, TNFR2 signaling takes place without subsequent TNFR1 stimulation via endogenous TNF. Activation of TNFR1 but not TNFR2 robustly upregulated A20 and TRAF1 in TNF-knockout macrophages (Fig. 4). TNFR2 stimulation, however, resulted in a strong enhancement of A20/TRAF1 expression, triggered by direct and selective stimulation of TNFR1 with low concentrations of human TNF (Fig. 4, lanes with 0.5 ng/ml TNF). Moreover, this enhancing effect was abrogated when cells were additionally treated with necrostatin-1 (Fig. 4).

## Discussion

**Fig. 5:**
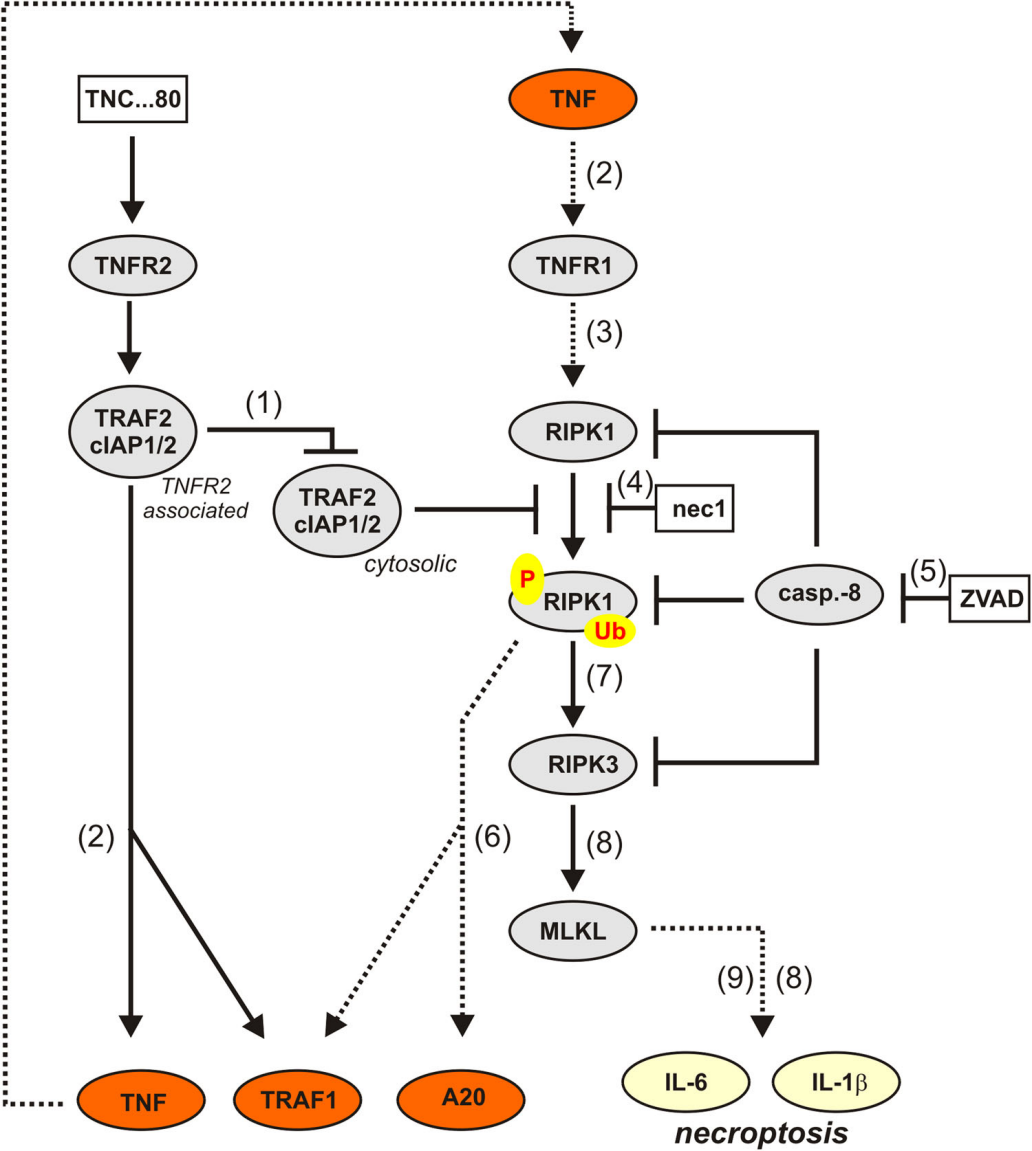
Model of TNFR2-triggered, necrostatin-1-sensitive TNFR1 signaling. We previously showed in caspase-inhibited macrophages that exclusive stimulation of TNFR2 results in depletion of TRAF-cIAP1/2 complexes (1), activation of the alternative NFκB pathway (2) and necroptosis (8). In this study, we show that TNFR2 in caspase-inhibited (5) macrophages in addition upregulates A20, TRAF1, IL-6, and IL-1β (6, 9) via TNF induction (2), and subsequent TNFR1 stimulation (3). Notably, although RIPK1 kinase activation is required for upregulation of all four targets (4), there are different requirements of the necroptotic signaling components RIPK3 and MLKL. Although A20 and TRAF1 are upregulated in the absence of RIPK3, MLKL and necroptosis (6), induction of IL-6 and IL-1β (9) is linked to necroptosis (8)

In a previous study, we found that stimulation of the non-death receptor TNFR2 alone is fully sufficient to induce necroptosis in caspase-inhibited macrophages^14^. In light of what is known about TNF-induced necroptosis, this finding was unexpected and could be traced back to the induction of TNF expression and subsequent activation of the well-established necroptosis-inducing receptor TNFR1^14^. Noteworthy, TNFR2 crucially contributes to necroptosis beyond triggering of TNF production by depletion of TRAF2-cIAP1/2 complexes sensitizing macrophages for TNFR1-induced necroptosis. As TNFR2-induced necroptosis in caspase-inhibited macrophages ultimately reflect gene inductive TNFR2 signaling, we analyzed gene induction upon exclusive exogenous stimulation of TNFR2 in more detail using the TNFR2-specific mouse TNF variant TNC-sc(mu)TNF80^21^. These investigations came up with two puzzling observations: first, TNFR2-induced expression of TNF and other NFκB-regulated factors was strongly enhanced by caspase inhibition (ref^14^. and Fig. 1). The relevance of caspase inhibition is not only unexpected as TNFR2 does not trigger caspase activation but also because it is well established that caspases inhibited NFκB signaling and protein translation^16,23^. Second, TNFR2-stimulated gene induction was dependent on the kinase activity of RIPK1. Again this is unexpected, because RIPK1 has so far not been implicated in TNFR2-induced NFκB signaling. The observed unusual features of TNFR2-triggered gene induction, however, perfectly match with the requirements of TNFR2-induced necroptosis. It appeared therefore possible that TNFR2-triggered gene induction similarly occurs via upregulation of TNF and subsequent TNFR1 stimulation. Indeed, TNFR2-stimulated gene induction was strongly diminished in TNF- and TNFR1- knockout cells but not fully abrogated (Fig. 2). Thus, a scenario appears possible in which weak but direct TNFR2-stimulated gene induction is enhanced by triggering auto-/paracrine TNF–TNFR1 signaling. Indeed, TNFR2 activates the alternative NFκB pathway independently from caspase inhibition and RIPK1 kinase activity^14^ what can explain the initial TNF/TNFR1-independent gene inductive activity required to trigger autocrine TNF–TNFR1 signaling. This simple model, however, cannot explain a basic observation we made in course of our experiments. In contrast to TNFR2-induced, TNFR1-mediated gene induction neither TNFR2-induced alternative NFκB signaling nor NFκB signaling triggered directly via TNFR1 require RIPK1 kinase activity. Thus, in caspase-inhibited macrophages, TNFR2 not only triggers TNFR1 signaling by upregulation of endogenous TNF expression but also basically changes the TNFR1 cell response pattern from RIPK1 kinase activity-independent gene induction and survival to yet, for TNFR1 unprecedented, RIPK1 kinase activity-dependent gene induction and necroptosis. It is tempting to speculate that this novel mode of TNFR1-mediated gene induction represents an epiphenomenon of necroptosis. To evaluate this possibility, we analyzed MLKL and RIPK3-deficient macrophages. As expected, these cells were resistant against necroptosis induction by TNFR2/ZVAD (Fig. 3a). Moreover, induction of IL-6 and IL-1β was also largely abrogated suggesting that the production of these two cytokines occurs as an epiphenomenon of TNFR1-driven necroptosis (Fig. 3c). More interestingly, however, upregulation of TRAF1 and A20 remained unaffected in TNFR2/ZVAD-stimulated caspase-inhibited MLKL- and RIPK3-knockout macrophages and was still blocked by necrostatin-1 (Fig. 3b). Thus, the novel RIPK1 kinase activity-dependent mode of gene induction by TNFR1, which is only evident in response to TNFR2 stimulation in caspase-inhibited macrophages, bifurcates upstream of RIPK3 from necroptosis (Fig. 5). As exclusive stimulation of TNFR1 fails to trigger RIPK1 kinase activity-dependent gene induction, TNFR2 obviously primes caspase-inhibited macrophages for this pathway. Indeed, in TNF-knockout cells where endogenous TNF cannot affect TNFR1 activity, TNFR2 treatment enabled TNFR1 to utilize a RIPK1 kinase-dependent mechanism in addition to the usual RIPK1 kinase-independent pathway to induce TRAF1 and A20 (Fig. 4).

The necroptosis-independent but RIPK1 kinase activity-dependent mode of gene induction in caspase-inhibited macrophages described in this study for the TNF–TNF receptor system is not without precedence. Just recently, it has been reported that lipopolysaccharide (LPS) triggers a similar mode of gene induction^24^. As TNF is a major target of LPS in macrophages, at first glance this could mean that LPS-induced RIPK1 kinase activity-dependent gene induction also involves the cooperative TNFR1–TNFR2 mechanism described in the present study. However, this appears not to be the case, because Najjar et al.^24^ observed unchanged ZVAD/LPS-induced gene induction in TNFR1-knockout macrophages. In macrophages, LPS signals via TLR4 and the TLR4-interacting adapter proteins TRIF and MyD88. Noteworthy, TLR4 recruits and activates RIPK1 via TRIF^24^ and triggers degradation of TRAF2 and cIAP1 via MyD88^10^. In context of ZVAD/LPS signaling, the TLR4-MyD88 and TLR4-TRIF axes may therefore cooperate similarly as TNFR2-dependent TRAF2-cIAP1/2 depletion and TNFR1-induced RIPK1 activation in context of TNC-sc(mu)TNF80-induced signaling. However, there are also yet not explainable differences between ZVAD/TNC-sc(mu)TNF80- and ZVAD/LPS-induced RIPK1 kinase activity-dependent gene induction. Although in the case of ZVAD/LPS gene induction required RIPK3 but not MLKL^24^, in the case of ZVAD/TNC-sc(mu)TNF80 stimulation both molecules were largely dispensable for induction of A20 and TRAF1 (Fig. 3b) but were both required for IL-1β induction (Fig. 3e). Thus, dependent on the stimulus and target gene considered, RIPK1 kinase activity-dependent gene induction in caspase-inhibited macrophages can occur in three variations: (i) RIPK3- and MLKL-independent (TNFR2-triggered A20/TRAF1 expression); (ii) RIPK3-dependent but MLKL-independent (LPS-induced gene expression); and (iii) RIPK3- and MLKL-dependent (TNFR2-triggered IL-1β expression). Future studies must now elucidate how the different ways of RIPK1 kinase activity-dependent signaling are created at the molecular level and whether, and if yes how, they are functionally interconnected.

The RIPK1 kinase activity-dependent mode of gene induction of TNFR1 requires caspase inhibition to become evident, although there was no obvious apoptosis induction in TNF-treated macrophages. Thus, it appears possible that low/tonic caspase activity, insufficient to trigger apoptosis, constitutively inhibit this mode of TNFR1 signaling. Another future challenge is therefore to identify the physiological conditions under which TNFR1-induced RIPK1 kinase activity-dependent gene induction gains relevance in macrophages and whether this mode of TNFR1 signaling is also operative in other cell types.

## Materials and methods

### Cells and reagents

Macrophages were differentiated from stem cell factor (SCF)-ER-Hoxb8 immortalized murine myeloid progenitor cells (MPCs). The latter were generated essentially as described by Wang et al.^25^ using femurs of C57Bl/6 wild-type mice and TNFR1-, TNFR2-, TNF-, MLKL-, and RIPK3-knockout mice and lentivirus with estrogen-regulated HoxB8 expression. The required estrogen-regulated expression plasmid 3HAERHBH-HoxB8 was a kind gift of Hans Haecker (St. Jude Children’s Research Hospital, Memphis, USA). The bones of RIPK3- and MLKL-knockout mice were kindly provided by Philip Jost (Technical University Munich) Immortalized MPCs were expanded in MPC medium (Optimem (first 3 days) or RPMI 1640 (after 3 days) supplemented with 10% (v/v) fetal calf serum (FCS), 1% (v/v) l-glutamine, 30 µM β-mercaptoethanol with 1 µM estradiol (Sigma) and 4% (v/v) SCF containing cell culture supernatant derived of SCF-producing CHO cells). MPC medium was changed two to three times per week. For macrophage differentiation, MPCs were washed three times with warm 10% FCS (v/v) supplemented phosphate-buffered saline (PBS). Cells (10 × 10^6^) were subjected to uncoated cell culture plates in macrophage differentiation medium (RPMI 1640 medium, 10% (v/v) FCS, 1% (v/v) l-glutamine, 30 µM β-mercaptoethanol, 10% (v/v) conditioned cell culture supernatant of macrophage colony-stimulating factor-producing L929 cells. Medium was replaced once after 3 days and after 2 additional days cells were collected by scraping with a rubber policeman and analyzed by flow cytometry for cell surface expression of CD11b and F4/80 to control for the success of macrophage differentiation. For viability assays and evaluation of cytokine induction, macrophages were seeded in 96-well plates (45 × 10^3^ per well) and were stimulated the next day. For western blotting and quantitative PCR (qPCR) experiments, macrophages were seeded in six-well plates (1 × 10^6^ per well).

Antibodies used in this study were from Miltenyi Biotec, Bergisch Gladbach, Germany (anti-CD11b rat IgG2b-PE, clone M1/70.15.11.5, #130-091-240; rat IgG2b-PE, #130-102-663; anti-mouse F4/80 human IgG1, clone REA126, #130-102-943; REA control antibody-PE, clone REA239, #130-104-613), Cell Signaling Technology, Beverly, MA, USA (anti-A20, rabbit IgG1, #5630; anti-p-RIPK1(Ser166), rabbit polyclonal IgG, #31122; anti-TRAF2, rabbit polyclonal IgG, #4724), Santa Cruz, USA (anti-TRAF1, rabbit polyclonal IgG, #sc-1831), Enzo (anti-RIPK3, rabbit polyclonal IgG, #ADI-905-242-100), BD Bioscience (anti-RIPK1, mouse IgG2a #610459), Abcam, Cambridge, UK (anti-p-RIPK3(S232), rabbit polyclonal, #ab195117) and NeoMarkers, Fremont, USA (anti-tubulin alpha, mouse IgG, #MS-581-P).

The pan caspase inhibitor ZVAD was purchased from Thermo Fisher Scientific Waltham, MA, USA (Bachem #N-1510.0025) and the RIPK1 inhibitor necrostatin-1 was from Biomol, Hamburg, Germany (#AP-309). Human TNF was a kind gift from Professor Daniela Männel (University of Regensburg, Germany). Production, purification, and properties of the agonistic murine TNFR2-specific nonameric murine TNF variant TNC-sc(mu)TNF80 have been described elsewhere^21^. In brief, HEK293 cells were transiently transfected with an expression plasmid encoding TNC-sc(mu)TNF80 and after 5–7 days supernatants were collected. By help of the FLAG tag contained in TNC-sc(mu)TNF80, the protein was purified from the pooled supernatants by affinity chromatography on anti-FLAG mAb M2 agarose according to the recommendations of the supplier (Sigma, Deisenhofen, Germany). Purified TNC-sc(mu)TNF80 were regularly controlled for potential LPS contaminations acquired during purification and/or storage using the Pierce^TM^ LAL Chromogenic Endotoxin Quantitation Kit (Thermo Fisher Scientific). If required, LPS was removed with the Pierce™ High Capacity Endotoxin Removal Resin.

### Quantitative PCR

Macrophages (1 × 10^6^) were stimulated with the reagents of interest for 7 h. After washing the cells twice with ice-cold PBS RNA was prepared with the RNeasy mini kit (Qiagen,Valencia, CA, USA) following the recommendations of the supplier. RNA (2 µg) were transcribed into DNA using the high-capacity cDNA reverse transcription kit from Applied Biosystems (Carlsbad, CA, USA) and mRNA levels were quantified using an ABI Prism 7900 sequence detector (Applied Biosystems) and TaqMan^TM^ gene expression assays (Applied Biosystems) for mouse *Cflar* (Mm01255578_m1), *Tnfaip3* (Mm00437121_m1), *Traf1* (Mm00493827_m1), *Tnf* (Mm00443258_m1), and Hprt1 (Mm00446968_m1). Reactions were performed in technical triplicates for each sample of an experiment. Expression levels of mRNAs were calculated by help of the SDS 2.1 software (Applied Biosystems) and were finally normalized to the housekeeping gene Hprt1.

### Western blotting

Macrophages were stimulated with the reagents of interest and were subsequently scraped into ice-cold PBS with a rubber policeman. Cells were spin down by centrifugation (2 min, Eppifuge full-speed) and resuspended in 4 × Laemmli sample buffer (8% (w/v) SDS, 0.1 M dithiothreitol, 40% (v/v) glycerol, 0.2M Tris, pH 8.0) supplemented with phosphatase inhibitor cocktail II (Sigma, Steinheim, Germany). Cell lysis was enhanced by sonification (ten pulses) and heating (5 min, 96 °C). Lysates were cleared from remaining insoluble debris by centrifugation (8 min, Eppifuge full-speed) and subjected to SDS-polyacrylamide gel electrophoresis. After separation proteins were transferred from the gel to nitrocellulose by wet blotting for 90 min. Remaining free binding sites on the nitrocellulose were blocked by overnight incubation at 4 °C in Tris-buffered saline with 0.1% (v/v) Tween 20 and 5% (w/v) dry milk. After washing once with TBST, the nitrocellulose membrane was incubated with the primary antibodies of interest (4 °C, overnight). After washing twice again in TBST, the nitrocellulose membrane was incubated with an appropriate horseradish peroxidase-conjugated secondary antibody (Dako, Hamburg, Germany) and the antigen–antibody complexes were finally detected by help of the ECL western blotting detection reagents and analysis system (Thermo Fisher Scientific).

### Cellular viability and ELISA assay

Macrophages were seeded in 96-well tissue culture plates (45 × 10^3^/well) in 100 µl culture macrophage differentiation medium. The following day, macrophages were stimulated in triplicates with the reagents of interest for 36 h. Finally, either cell viability was determined using the MTT assay or upregulation of IL-6 and Il-1β production was measured by enzyme-linked immunosorbent assay (ELISA) of supernatants (R&D: mouse IL-1β/IL-1F2 DuoSet, #DY401; BD Bioscience: OptEIA mouse IL-6 ELISA Set, #555240). In each cell viability experiment, one triplicate of cells was treated with a cytotoxic cocktail containing 1 µg/ml Fc-CD95L, 20 µM CHX, and 1% (w/v) sodium azide, which triggers complete cell death. The triplicate of cells challenged with the cytotoxic mixture defined 0 % viability and the triplicate of untreated cells defined 100% viability. All other viability measurements were normalized against these values. The averages of the technical triplicates were considered as a data point for further statistical evaluation (one-way analysis of variance Bonferroni’s multiple comparison test function; GraphPad Prism5 software from GraphPad software, La Jolla, CA, USA).
